# Essential Oils of *Artemisia frigida* Plants (Asteraceae): Conservatism and Lability of the Composition

**DOI:** 10.3390/plants12193422

**Published:** 2023-09-28

**Authors:** Svetlana V. Zhigzhitzhapova, Elena P. Dylenova, Bato V. Zhigzhitzhapov, Danaya B. Goncharova, Zhargal A. Tykheev, Vasiliy V. Taraskin, Oleg A. Anenkhonov

**Affiliations:** 1 Baikal Institute of Nature Management, Siberian Branch, Russian Academy of Sciences, 670047 Ulan-Ude, Russia; zhig2@yandex.ru (S.V.Z.); zhbat120401@gmail.com (B.V.Z.); danaydomi5@gmail.com (D.B.G.); gagarin199313@gmail.com (Z.A.T.); vvtaraskin@binm.ru (V.V.T.); 2 Institute of General and Experimental Biology, Siberian Branch, Russian Academy of Sciences, 670047 Ulan-Ude, Russia; anen@yandex.ru

**Keywords:** arid territories, terpenoids, 1,8-cineol, camphor, borneol, climate, phenological phase, elevation above sea level

## Abstract

Plants of arid regions have adapted to harsh environments during the long span of their evolution and have developed a set of features necessary for their survival in water-limited conditions. *Artemisia frigida* Willd. (Asteraceae) is a widely distributed species possessing significant cenotic value in steppe ecosystems due to its high frequency and abundance. This study examines different patterns of formation of essential oil composition in *A. frigida* plants under the influence of heterogeneous factors, including climate and its integral characteristics (HTC, C_extr_, SPEI and others). The work is based on the results of our research conducted in Russia (Republic of Buryatia, Irkutsk region), Mongolia, and China, from 1998 to 2021. A total of 32 constant compounds have been identified in the essential oil of *A. frigida* throughout its habitat range in Eurasia, from Kazakhstan to Qinghai Province, China. Among them, camphor, 1,8-cineol and bornyl acetate are the dominant components, contained in 93–95% of the samples. Among the sesquiterpenoids, germacrene D is the dominant component in 67% of the samples. The largest variability within the composition of the essential oils of *A. frigida* is associated with significant differences in the climatic parameters when plants grow in high-altitude and extrazonal conditions.

## 1. Introduction

Currently, the issue of biota response to ongoing climate warming, along with the back-reaction between “biota” and “climate”, is at the cutting edge of global biology and ecology. There is no doubt that climate change is happening and will continue to happen in the foreseeable future. Vegetation is forced to respond to biotically significant changes in climate, whether they are significant once, are episodic oscillations, or sustainable long-term trends. Therefore, a key scientific problem is the study of mechanisms of plant adaptation to the conditions of short-term or long-term exposure to adverse environmental factors.

During the current climate warming, arid territories are occupying increasingly extensive areas. Plants in arid regions have adapted to harsh environmental conditions during the long span of their evolution, and have developed a set of specific morphological, physiological and phenological features necessary for their survival in water-limited conditions. The study of these plants growing in natural populations will allow to demonstrate the stability of the plant locus with respect to drought [1].

*Artemisia frigida* Willd. (Asteraceae) has significant cenotic value as dominant or co-dominant in the composition of the cryophyte, true and desert steppes of Siberia, the Volga region, Kazakhstan, Central Asia, Mongolia and North America. The high plasticity of the species, as well as its ability of rapid vegetative reproduction through the development of creeping and quickly rooting aboveground shoots, determine the competitiveness of *A. frigida* plants when pasture degradation is intensifying [2]. *Artemisia frigida* plants are heat-resistant, being able to survive in environments comparable to habitats of typical desert plants with temperatures reaching 56–57 °C [3].

In conditions of high air temperatures and aridity of the territory, volatile terpenoid compounds act as the first line of defense for the photosynthetic apparatus. Thus, the fumigation of plants with monoterpenes increases their thermal resistance. Monoterpenes, as well as many volatile sesquiterpenes found in plants, quickly react with reactive oxygen species (ROS) [4], thereby protecting the membranes of plant cells from oxidative damage. Sesquiterpenes can mitigate damage even more effectively than isoprene and monoterpenes. For example, the sesquiterpene (E)-β-caryophyllene is 43 times more reactive with ozone than the monoterpene limonene. Two other sesquiterpenes, (E)-β-farnesene and (E)-α-bergamotene, have a high potential for protecting against abiotic stress [5]. The protective mechanism is associated both with direct reactions of terpenoids with oxidants, either intracellularly or at the boundary between the leaf and the atmosphere/ boundary layer, stabilizing the membrane, and with indirect changes in ROS signal transduction [6]. Plants emit an enormous number of terpenoids annually, reaching huge quantities (500 teragrams) [7]. Climate changes affect terpenoid emissions and have significant consequences for the structure and functioning of the biosphere [8]. In addition to high temperatures, terpenoid emissions can also be induced by herbivore feeding, pathogen attack, radiation, salt stress and other abiotic stresses [9,10].

Terpenoid compounds are obtained from plants in the form of complex mixtures known as essential oils (EOs). EOs possess antibacterial, antifungal, herbicidal and other properties, and therefore have found wide applications in the pharmaceutical, food, agricultural, cosmetic and healthcare industries [11,12]. EOs are complex mixtures of both native components and artifact compounds that inevitably form because of such processes as storage, drying of plant material, extraction and analysis. Their composition is related to many variables, such as cultivar genotype [13], the geographical area of production [14], harvest year [15], and extraction system [16]. When obtaining EOs under the same conditions of storage and drying of raw materials, and using the same method of extraction and analysis of EOs under strictly defined parameters, we can observe the influence of genetic and epigenetic factors on the composition.

The aim of this study is to determine the patterns of the formation of the composition of the essential oil of *Artemisia frigida* Willd. plants, and to give possible reasons for its conservatism and lability.

## 2. Results and Discussion

### 2.1. Composition of Essential Oils of Artemisia frigida (Based on Plants Growing within the Territory of Buryatia (Russia) and Qinghai (China) from 2016–2021

The isolated EOs were light, mobile liquids of a pale yellow color with a yield ranging from 0.3 to 1.0% (*v*/*w*). A total of 179 compounds were identified in the samples of the essential oil, with the majority being monoterpenoids and sesquiterpenoids (Supplementary Materials, Table S1).

For data analysis convenience, the entire variety of identified compounds were divided into four groups, based on their frequency of occurrence in the 27 tested samples: (1)—components found in 75% or more of the samples; (2)—in 50–74% of the samples; (3)—in 26–49% of the samples; and (4)—in 25% or less of the samples.

The first group was represented by 21 compounds. Only 1 compound among these, bornyl acetate, was found in all 27 samples; 9 compounds (γ-terpinene, para-cymol, terpinen-4-ol, α-terpineol, camphor, α-pinene, germacrene D, caryophyllene oxide, spathulenol) were found in 26 samples. Another 11 compounds were found in 21–25 samples. The mass fractions of components forming the first group ranged from 26.25% to 91.17%, meaning that they were the dominant components of *A. frigida* EOs. Among them, three compounds—camphor, borneol and 1,8-cineol—were part of the top five dominant components in 81% of the studied samples. Less frequently among the dominant compounds (in at least one of the samples), bornyl acetate, α-terpineol and terpinen-4-ol from this group were observed. There were also such compounds from this group as linalool, (E)-nerolidol, α-copaene, β-pinene, salvial-4(14)-en-1-one, and β-myrcene, which were not found as dominant in any of the studied samples (Supplementary Materials, Table S2).

The second group consisted of 19 compounds. Such components as *para-trans*-menth-2-en-1-ol, myrtenol, bicyclogermacrene, and silphiperfol-6-en-5-one were identified in 20 out of 27 samples. The remaining 15 compounds were found in 14–19 samples. The total mass fraction of compounds of the second group was 0.96–29.34%. The content of each of the components, with the exception of myrtenol, selinene, beta- and isocaryophyllene in individual samples, did not exceed 2–3%.

The third group was formed of 38 compounds. They were identified in 7–13 samples, where their total content ranged from 1.52% to 22.40%. The proportion of individual compounds in this group did not exceed 1–2%, except for (E)-β-farnesene, (Z,E)-α-farnesene, neointermedeol, and cis- β- p-mentha-6,8-dien-2-ol in certain samples.

The fourth group was the most numerous (101 components). However, fifty compounds were found only in one of the samples, and only five (nonanal, nerol, trans-chrysanthenol, trans-sabinene hydrate, 7-epi-silphiperfol-5-ene) were identified in six samples. The total proportion of components in this group varied from 0.72% to 59.93%. The mass fraction of most compounds in this group did not exceed 1–2%, except for two cases: the proportion of α-terpineol acetate in the sample “BURi 30.08.2017” was 6.30%, and that of α-thujone in the sample “OLNs 05.08.2017” was 10.52%.

Among the studied samples, the sample from China «CHN, 2016 4333 m» and four samples from Buryatia “BUR 1343 m 17.08.2016”, “BURss 24.08.2016”, “BURroad 19.08.2021”, and “BURe 25.08.2016” were different from others in terms of the component composition of EOs. For example, the sample “CHN, 2016 4333 m” contained only caryophyllene (17.78%) among the top five dominant components from the first group, while the rest of the dominants were from the fourth group: methyl eugenol (16.16%), caryophylla-3(15),7(14)-dien-6-ol (14.36%), β-sesquiphellandrene (8.91%) and α-fenchene (4.42%). The dominant components of the sample “BUR 1343 m 17.08.2016” were spathulenol (14.05%), germacrene D (5.58%), caryophyllene oxide (3.54%) from the first group, (E)-β-farnesene (12.88%) from the third group, and acorenone (5.25%) and (2E, 6E)-farnesol (3.43%) from the fourth group.

The “BURss 24.08.2016” sample was also different from the others, since it contained compounds from the first group—para-cymol (12.6%), as well as spathulenol in a noticeable amount (7.97%). In addition, the dominant components of this sample included a component from the third group, (E)-β-farnesene (7.97%). The component composition of the first six dominant components of the essential oil of the sample “BURroad 19.08.2021” included compounds from the first group (germacrene D 13.01%, spathulenol 9.84%, and caryophyllene oxide 6.94%), the second group (isocaryophyllene 10.12%, and β-selinene 5.33%) and one compound from the third group (β-elemene 5.33%). At the same time, “BURe 25.08.2016” contained isocaryophyllene (9.96%) and approximately equal amounts (5–6%) of common compounds found in most samples, such as 1,8-cineol, borneol, germacrene D, spathulenol and caryophyllene oxide.

The differences in the composition of the dominant components in the samples “CHN, 2016 4333 m” and “BUR 1343 m 17.08.2016” can be explained by the growth of plants in areas of high altitudes. The composition of the sample “BURroad 19.08.2021” components may be related to the fact that plants grew close to the federal road R258 “Baikal”, with intensive traffic. The reasons for the differences in the composition of EOs in other samples are currently unclear. In any case, it is obvious that the composition of components and the content of their EOs are changeable under the influence of different factors. In addition, let us consider some of the factors that influence the composition of EOs of *Artemisia frigida* in more detail.

### 2.2. Composition of Essential Oils of A. frigida during Ontogenesis and Phenological Development of Plants

It has been established that changes occur in the biosynthesis of plants during ontogenesis in *A. frigida*. Indeed, it has been shown with potted crops that during the first year of plant life, changes occur in nitrogen metabolism. Specifically, the nitrogen content in shoots and roots decreases throughout the vegetative period [17]. However, the genetic basis of these changes in biosynthesis during ontogenesis is currently unknown. An interesting example of this is the encoding of the biosynthesis of a large number of sesquiterpenes in patchouli plants with only five genes, which may be indirect evidence of the importance of epigenetic mechanisms in the biosynthesis of terpenoid compounds in plants [18]. The first step towards differentiating the genetic and epigenetic nature of the components of EOs may be the study of their dynamics during plant development, as it is known that the quantity and composition of EOs change not only in space but also during plant ontogeny [19,20]. Unfortunately, there are no data on the dynamics of the qualitative and quantitative composition of the essential oil at all stages of *A. frigida* ontogeny. Only the composition of the essential oil in the latent and generative phases of age development has been studied.

The latent phase of plant ontogeny is represented by fruits. In *A. frigida*, the fruit seeds contain EOs that act as anti-attractants for herbivore feeding, inhibitors of seed germination, sprout growth and reproduction of other species [21,22]. The same components identified in the EOs obtained from the aboveground parts are also found in the composition of the fruit EOs. The main components of the fruit EOs are compounds with allelopathic activity: camphor (22.58%), borneol (22.46%), 1,8-cineol (25.11%), terpinene-4 (4.79%) and bornyl acetate (3.57%). The content of the remaining components does not exceed 1.5% (Table 1).

Interestingly, unlike the aboveground parts of adult *A. frigida* plants in the generative phase of ontogeny, most of the oil in the fruits consists of monoterpenoids, which are characterized by greater volatility (91.33%). In our opinion, monoterpenoids facilitate the survival of the fruits, including protection against pests such as invertebrates (insects and others) and fungi and bacteria, as more volatile compounds interact more quickly with the surrounding environment during the early stages of seed germination and require less energy for their biosynthesis.

The composition of the essential oil changes during different phases of plant phenological development. For example, in the study [23], it was shown that as the Lavandula × intermedia flower develops, the camphor content in the essential oil remains unchanged, but the concentration of borneol (a precursor to camphor) in the tissues increases. However, nowadays there is no direct molecular link between plant development and terpene biosynthesis that has been revealed [18].

The compositions of the EOs of *A. frigida* plants collected in the same populations but in different phenological phases were studied. The EOs were isolated from plants collected in three populations of the Ivolginsky district of the Republic of Buryatia in 2021: steppe (“BURi 27.07.2021”, “BURi 20.08.2021”, “BURi 17.09.2021”), roadside (“BURroad 19.08.2021”, “BURroad 17.09.2021”) and hilltop (“BURhill 27.07.2021”, “BURhill 19.08.2021”). The plants collected in July were in the budding phase, in August the flowering phase, and in September the fruiting phase.

During the transition from one phase to another, the yield of essential oils did not change, but the composition slightly changed. Compounds in the first group (except β-pinene, β-myrcene) were found in all stages of plant development, and only their proportion in the essential oil changed. However, there was no specific trend of decrease or increase in the transition from one phenophase to another, meaning that the direction of change was different for each population. Monoterpenoids such as β-pinene and β-myrcene were identified in the flowering (“BURroad 19.08.2021”, “BURhill 19.08.2021”) and fruiting phases (“BURroad 17.09.2021”, “BURi 17.09.2021”). Compounds of the second group were also found in all phenophases, except myrtenyl acetate. The latter was only identified in the sample “BURi 20.08.2021”. This sample also, unexpectedly, had a high content of the monoterpenoid alcohol myrtenol (4.27%). In the sample “BURhill 27.07.2021”, silphiperfol-6-en-5-one (4.43%) and isocaryophyllene (7.11%) differed significantly in content compared to other samples, and the latter was also found in significant amounts in the sample “BURroad 19.08.2021” (10.12%). Compounds in the third group can be found in all phenophases (β-elemene, pentadecanal, etc.) as well as in specific phenophases (for example, β-copaene in the budding phase, and cabreuva oxide B in budding and flowering). Some of them (silphiperfol-6-ene, aristolochene, 4,5-di-epi-, teointermedeol, β-elemene, etc.) may have a content higher than 1%, reaching, for example, 5.33% for β-elemene. The majority of components in the fourth group were found in only one sample. Mintoxide, *trans*-sabinene hydrate and eugenol were identified in all phenophases. In the budding and flowering phases, 1,8-, 2,3-dehydro-cineol, and chrysanthenone were detected, and in the flowering and fruiting phases salviadienol was found. Davanafuran isomer and silphiperfola-4,7(14)-diene were detected in the budding and fruiting phases (Supplementary Materials, Table S3).

**Figure 1:**
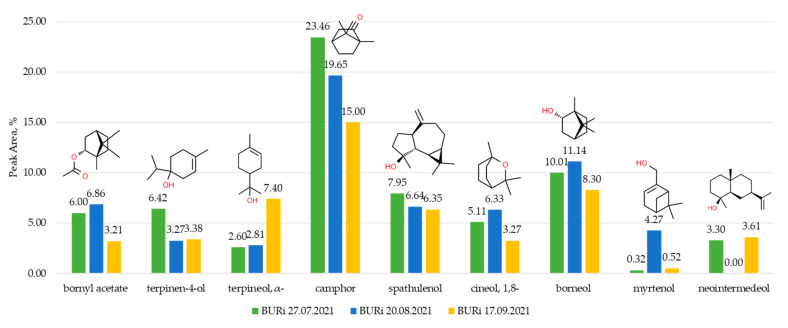
Content of major components of EOs of *A. frigida* plants during budding phase (green), flowering phase (blue) and fruiting phase (yellow).

The dynamics of the content of major components (with a content of 3% and more) of the essential oil from budding to fruiting was considered using a specific steppe population as an example. The main components included compounds from the first group: bornyl acetate, terpinen-4-ol, α-terpineol, camphor, spathulenol, 1,8-cineol and borneol, as well as one component each from the second (myrtenol) and third (neointermedeol) groups. The changes in the content of camphor, spathulenol and borneol were insignificant. However, the proportion of terpinen-4-ol decreased by half during the transition from budding to flowering, while α-terpineol increased 2.6 times during fruiting compared to other phenophases. The changes in the content of components from the labile part of the *A. frigida* essential oil were more significant. For example, the content of myrtenol increased by 8.2–13.3 times during the flowering phase. Neointermedeol was not detected during the flowering phase, while its proportion was approximately the same during the budding and fruiting phases (3.30–3.61%) (Figure 1).

As for the trends in the content of individual compounds in different phenophases, four patterns can be identified, three monomodal (Nos 1–3) and one bimodal (No 4): (1) the content of the compound is highest during the budding phase and then gradually decreases or remains at the same level (camphor, spathulenol, and terpinen-4-ol); (2) the content reaches its peak during the flowering phase (bornyl acetate, 1,8-cineol, borneol, and myrtenol); (3) the content of the compound is highest during the fruiting phase (terpineol, α-); and (4) the compound is present during the budding and fruiting phases but absent during flowering (neointermedeol). The presence of different patterns in the essential oil content during phenological development demonstrates the complexity of adaptive mechanisms.

Thus, the composition of the essential oil changed during plant development, particularly during phenological development. Compounds from the first and second groups were found in almost all phenophases, but their content in the essential oil varied. They formed a constant part of the essential oil, but no trends in the dynamics of their content were observed. It can be assumed that the formation of compounds in the constant part of the essential oil is predetermined at the genetic level, and thus this set is an immanent property of the species. On the other hand, differences in the composition of compounds from the third and fourth groups in different phases of plant development can be explained by the formation of new functional organs in which additional substances are synthesized [24]. Their formation might be associated with the species’ biosynthetic reaction to certain conditions, which accordingly has an epigenetic nature, as well as with the transformations of native compounds during the processes of drying, extraction and analysis.

### 2.3. Composition of Essential Oils of A. frigida from the Same Population Depends on the Weather Conditions in Different Years

**Figure 2:**
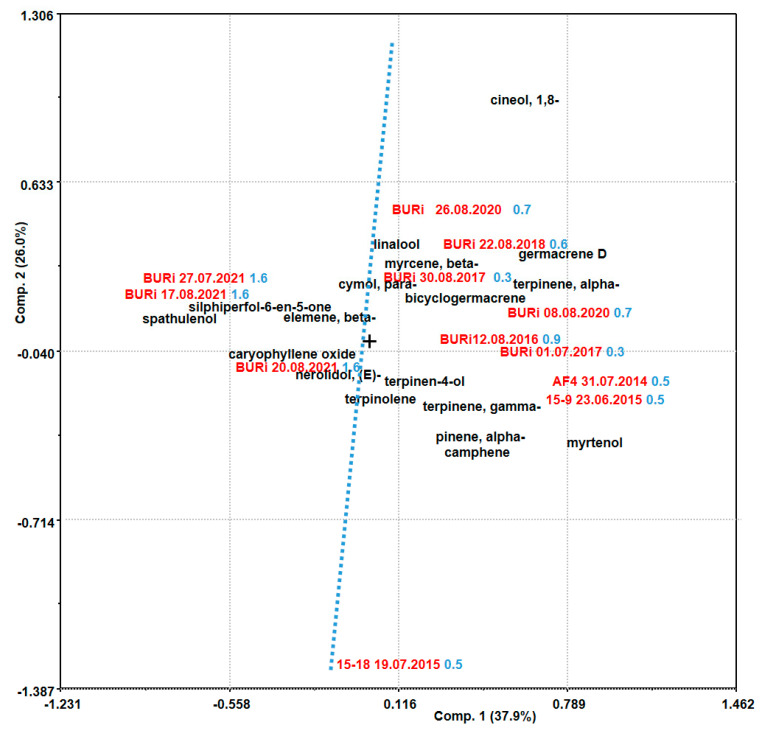
Biplot (PC1–PC2) of data on the content of constant compounds in the EOs of *Artemisia frigida* Willd. plants from the same population (Russia, Republic of Buryatia, and Ivolginsky district) in different years (2014–2021) (Principal Component Analysis). Samples “AF4 31.07.2014”, “15–9 23.06.2015” are previously published data [25]. Values of the hydrothermal coefficient are displayed in blue, sample codes—red.

The differences in the compositions of EOs obtained from plants of the same population at different phenophases are balanced compared to the differences between the compositions of EOs of the same population in different years, i.e., under different weather conditions. Thus, on the biplot (PC1–PC2), the projections of samples of EOs were divided into two parts. One part was grouped, regardless of the different phenophases of the samples, of samples from 2021, while the other part consisted of samples from other years (Figure 2). Indeed, the year 2021 significantly differed from others in terms of temperature and moisture conditions (hydrothermal coefficient—HTC 1.6), being more humid. In contrast, the years 2014–2020 were drier (HTC 0.3–0.9). The distance between samples collected in June and July 2015 (“15–9”, “15–18”) and July and August 2017 (“BURi 01.07.2017”, “BURi 30.08.2017”) can be explained by differences in climatic parameters from the beginning of vegetation to the date of collection. For example, in 2015, the average daily temperature from May to the date of plant collection (average t_veg._) for the “15–9” sample was 13.8 °C, while for “15–18” it was 16.1 °C. The cumulative rainfall in May until the date of plant collection (∑R_veg_) for the “15–9” sample was 29.1 mm, while for “15–18” it was 62.5 mm. Correspondingly, the extremeness coefficients (K_extr.veg._) differed almost twofold (0.5 and 0.3, respectively). In contrast, in 2021, temperature fluctuations and precipitation were more evenly distributed; climatic parameters (average t_veg_., ∑R_veg_) did not differ significantly at different collection times, and the extremeness coefficient (K_extr.veg._) from budding to fruiting had a value of 0.1 (Supplementary Materials, Table S4).

Correlation analysis showed a high (correlation coefficient values from 0.7 to 0.9) and very high (from 0.9 to 1.0) degree of association between the values of climatic parameters and some components of the essential oil. For example, the correlation coefficient values (0.92–0.98) between HTC values and the content of spathulenol, caryophyllene oxide and silphiperfol-6-en-5-one indicated a very high positive correlation between them. The values of average t_veg_. showed a high positive correlation with the content of borneol and a negative correlation with silphiperfol-6-en-5-one. The values of ∑R_veg_ positively correlate with the content of spathulenol and caryophyllene oxide, while K_extr.veg._ correlated positively with α- and γ- terpinene (Supplementary Materials, Table S5). Thus, the mass content of compounds from the first and second groups in the EOs of plants from the same population depends on the weather conditions in the year of raw material collection.

### 2.4. Composition of Essential Oils of A. frigida from Different Populations in Buryatia Depends Not Only on Climatic Conditions

**Figure 3:**
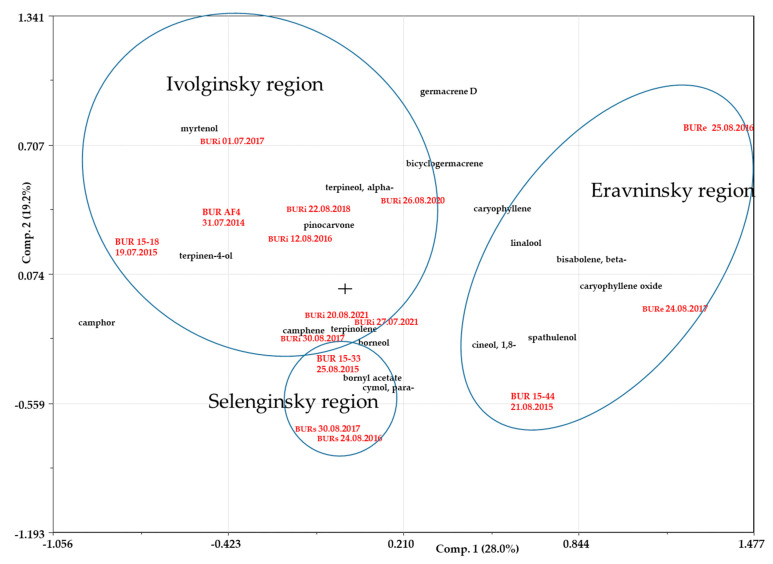
Biplot (PC1–PC2) of multi-year data on the content of constant compounds in the EOs of *Artemisia frigida* Willd. plants collected in the Ivolginsky, Selenginsky, and Eravninsky districts of Buryatia (Principal Component Analysis). Sample codes are displayed in red color, districts—blue circles.

PCA analysis was conducted on data obtained from other populations (in Selenginsky and Eravninsky districts) and compared with the EOs of plants growing in the Ivolginsky district. The differences in the composition of EOs of plants collected in different years were less pronounced than the differences between the compositions of EOs of plants from different locations (Figure 3).

The samples collected in the Eravninsky district differed the most. They were characterized by relatively low content of camphor (up to 5.5%) and noticeable amounts of certain sesquiterpenoids (β-bisabolene, caryophyllene, caryophyllene oxide, and spathulenol). In contrast, the composition of EOs from plants in the Ivolginsky and Selenginsky districts differed to a lesser extent. EOs of samples from the Selenginsky district contain higher amounts of cymol and para- and bornyl acetate compared to samples from the Ivolginsky district.

Linear regression models show that only the content of trans-carveol, para-, trans-menth-2-en-1-ol, camphor and bornyl acetate in the EOs has been changed under the influence of climatic parameters. For example, the content of trans-carveol was influenced by the climatic extremeness of the year. Among the dominant components, the content of camphor in the essential oil linked to changes in climatic parameters (Table 2).

The obtained results showed that the influence of climatic factors on the composition of EOs in plants was varying, which was also shown for plants of the genus *Nepeta* [26] and *Lavandula* [27]. Significant differences in the composition of EOs of plants from the Eravninsky district compared to the Selenginsky and Ivolginsky districts may be related to the fact that negative values of the average annual temperature lead to the existence of permafrost in the soils of the Eravninsky district, where permafrost has discontinuous distribution. In contrast, the Selenginsky and Ivolginsky districts belong to the zone of the only isolated distribution of permafrost. Thus, plants in the Eravninsky district experience a greater influence of low soil temperatures, which in turn is reflected in the composition of EOs. There is information in the literature about the influence of soil type [28] and substrate composition [29], but there are no data on the effect of soil temperature or permafrost on the composition of the essential oil, although the soil temperature affects the content of secondary metabolites in the plant. For example, a higher maximum soil temperature resulted in lower phenol content, while a higher minimum soil temperature and shorter harvest age increased the total flavonoids in Java tea (*Orthosiphon aristatus* B.) [30].

### 2.5. Composition of Essential Oils in Plants of A. frigida Confined to “Typical” and “Atypical” Habitats

The analysis of the composition of *A. frigida* EOs from 1998 to 2021 presented in this study and in previously published works [25,31,32,33], as well as literature data from different parts of the range: Siberia [34,35,36], Kazakhstan [35,37], Mongolia [38], Canada [39] and China [40], showed that a total of 283 compounds were identified. Camphor, 1,8-cineol and borneol were the dominant components in 93–95% of the samples, and among sesquiterpenoids, germacrene D was identified as a dominant component in 67% of the samples. The constant part of the EOs was represented by 32 components (Supplementary Materials, Table S6). It included all compounds in the first group of *A. frigida* EOs from the flora of Buryatia collected from 2016 to 2021 (Supplementary Materials, Table S2), except for salvial-4(14)-en-1-one and seven compounds from the second group: myrtenol, bicyclogermacrene, trans-carveol, humulene, β-selinene, para-cis-menth-2-en-1-ol, terpinolene, as well as some compounds from the third group: sabinene, trans-sabinene hydrate, β-elemene, α-elemene, and pinocarvone.

**Figure 4:**
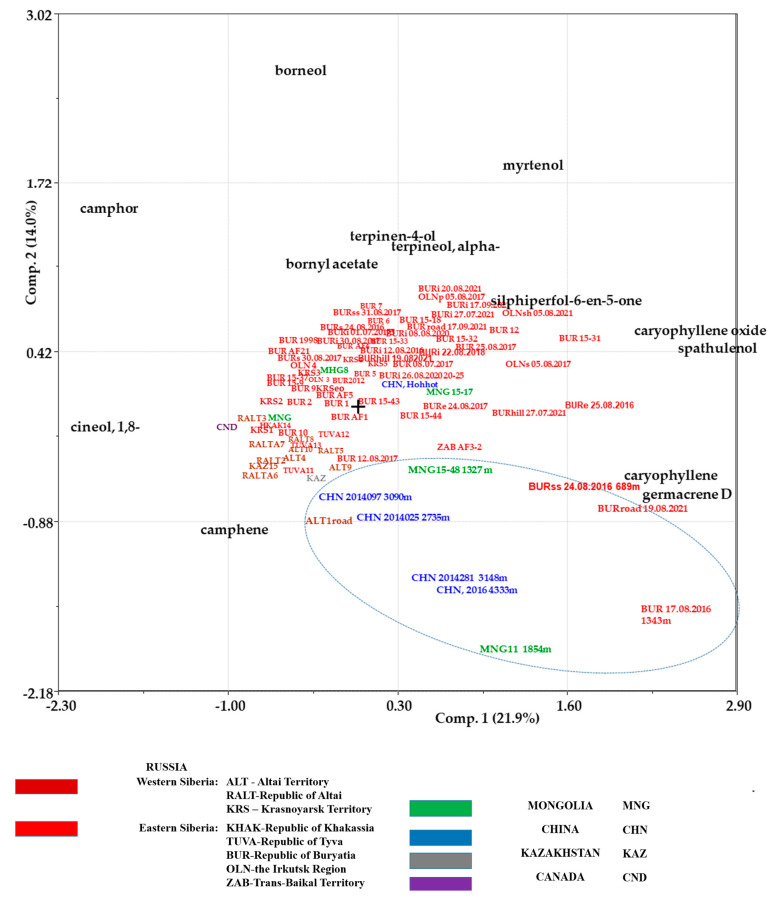
Biplot (PC1–PC2) of data on the content of constant compounds in the EOs of *Artemisia frigida* Willd. plants from different regions of the world (Principal Component Analysis). Designation “abbreviation and serial number”, “abbreviation and code”, “abbreviation, year”— published data, including own data from Russia (Republic of Buryatia, Irkutsk region) and Mongolia. Only designated with a code and collection date—data of 2016–2021.

Previously, it was shown for *Artemisia vulgaris* L. [41], *A. annua* L. [42], and *A. jacutica* Drob. [43] that under the influence of macroclimatic conditions, distinct “western” and “eastern” chemotypes of EOs were formed, which differed in their component composition. In the case of *A. frigida*, chemotypic differentiation was not observed, as there was no clear discrepancy between the essential oil samples obtained from “western” and “eastern” populations. In general, according to the PCA analysis in the case of *A. frigida*, most samples, regardless of their place of growth, did not show significant differences in composition. Nevertheless, there were 13 components that were most noticeably linked to climatic conditions, which was manifested in their greatest contribution to the distribution of samples on the biplot (Figure 4).

It can be noted that the majority of “western” samples were characterized by a relatively high content of monoterpenoids—camphor, 1,8-cineol and camphene. And for most “eastern” samples, in addition to monoterpenoids—bornyl acetate, terpineol-4, α-terpineol, and myrtenol—sesquiterpenoids such as spathulenol and caryophyllene oxide were presented in noticeable concentrations. However, even these features are not enough to allow for the identification of specific chemotypes. The reason may lie in the biological characteristics of *A. frigida*, which often grows on sandy and stony soils with relatively sparse vegetation cover [44]. Meanwhile, *A. frigida* plants from Western and Eastern Siberia are in similar conditions in terms of heat and moisture supply in their habitats. Thus, the values of hydrothermal coefficient (HTC) calculated from meteorological data from Western and Eastern Siberia ranged from 0.5 to 1.4, i.e., from “very arid” to “humid” conditions in both regions (Table 3).

However, 10 samples of EOs of plants growing in areas where climatic parameters differed from typical ones formed a single locus (“atypical” group, Figure 4). The differences were associated with the influence of high altitude, as well as the proximity of roads, or increased insolation. This locus was formed by plant samples from China, growing at altitudes of 2735–4333 m, Mongolia, growing at 1327 m and 1854 m, Buryatia, growing at 1343 m above sea level (a.s.l.), and plants growing near roads (“ALT1road”, “BURroad 19.08.2021”), as well as a sample of plants, “BURss 24.08.2016”, located on the southern slope of the Soldatsky Ridge in Buryatia.

If the differences in the composition of EOs in plants due to the influence of altitude (which determines climatic parameters) and the proximity of roads do not require any special explanations, then the influence of the slope exposure, which is the habitat of the plants, should be considered in more detail. Essential oil samples “OLN4”, “BUR10” [25] and “BURss 24.08.2016” were obtained from plants collected on slopes of different exposures. In the sharply continental climate of semiarid regions, including Transbaikalia and the Cis-Baikal area, the ecological conditions on different slopes differ significantly. Southern slopes receive more heat during the vegetative period. On southern insolated slopes the evaporation is much stronger, and plant communities experience a moisture deficit [45,46,47,48]. Thus, it is confirmed again that the composition of essential oil is determined by climatic parameters, in particular the ratio of heat to moisture.

All samples of the “atypical” group contained β-myrcene, terpinen-4-ol and germacrene D. If the content of terpinen-4-ol was lower than in the EOs of “typical” habitats, then the content of β-myrcene and germacrene D could reach significant levels. For example, the content of β-myrcene in the “MNG11 1854 m” sample was 7.90%, and that of germacrene D in samples “ALT1road” and “BURroad 19.08.2021” was 4.70% and 13.97%, respectively; in the sample “MNG11 1854 m” it was 20.30%. In 5–9 out of 10 samples, components were present in the EOs in noticeable concentrations. These include bicyclogermacrene (up to 2.68%), α-pinene (up to 5.90%), β-selinene (up to 6.72%), caryophyllene oxide (up to 6.94%), linalool (up to 10.31%), (E)-β-farnesene (up to 12.88%), spathulenol (up to 14.05%), 1,8-cineol (up to 22.88%), and camphor (up to 33.10%). At the same time, a number of components have been identified in half or more of the samples in this group, but the content of each of them did not exceed 1%: γ-terpinene, terpinolene, α-terpineol, (E)-nerolidol, caryophyllene, α-terpinene, camphene, bornyl acetate, β-pinene, α-copaene, and sabinene. Limonene, artemisia ketone, α-phellandrene, cis-davanone, β-elemene, and α-bisabolone oxide A were found in 3–4 samples, but their content was high. For example, in the samples “CHN2014281 3148 m”, “CHN2014097 3090 m”, and “CHN2014025 2735 m”, a significant proportion belonged to α-bisabolone oxide A (6.01%, 5.40%, and 24.25%, respectively). Acorenone was found only in two samples, and its content was significant: in “Oka 1343 m 17.08.2016” it was 5.25%, and in “CHN, 2016 4333 m” it was 8.90%. Chrysanthenone, α-thujone, α-bisabolol oxide B, and neointermedeol were also identified in only two samples, but only in one of them in large quantities. For example, the content of α-bisabolol oxide in the sample “CHN2014281 3148 m” reached 20.37%. In one of the samples of EOs of plants growing in high-altitude areas, components with a relatively high content were identified: methyl chavicol (4.30%) in the sample “MNG11 1854 m”, and α-farnesene (4.42%), (E)-methyl cinnamate (16.16%) and copaborneol (17.78%) in the sample “CHN, 2016 4333 m”. Thus, the composition of the dominant components of the EOs in this group of samples was different, especially in plants from high-altitude populations.

Samples from plants growing near the road (“ALT1road”, “BURroad 19.08.2021”) were most similar in composition and content of dominant components to the samples from “typical” habitats. Comparative analysis of the composition of EOs from *A. frigida* in “typical” and “atypical” habitats showed that 22 compounds were identified in half or more of the samples from both groups. These included camphor, 1,8-cineol, germacrene D and others. Some compounds found in the majority of EOs from “typical” habitats were found in fewer samples from “atypical” habitats. Borneol was found in all samples from “typical” habitats and in only 4 out of 10 samples from “atypical” habitats. Myrtenol was found in only one of the samples from “atypical” habitats. On the other hand, (E)-β-farnesene was found in 7 out of 10 samples from “atypical” habitats, while it occurred in only 20% of the EOs from plants in “typical” habitats. Among the other 261 components identified in less than half of the samples, 109 were found only in the EOs from “typical” habitats, 27 were found in “atypical” habitats, and 112 were common to plants from both groups. For example, α-bisabolol was specific to 18% of the samples from “typical” habitats, and yomogi alcohol was found in 3 out of 10 samples from “atypical” habitats, while trans-piperitol was identified in the EOs of both groups.

### 2.6. Conservatism s and Lability of the Composition of A. frigida Essential Oils

Based on the abovementioned, it was possible to distinguish the “core” of the *A. frigida* essential oil composition, which referred to those components that were formed as part of the genetic programs of terpenoid biosynthesis, regardless of the plant’s habitat. A total of 24 such components formed the “core”. However, the content of some of these components in the essential oil could vary during plant development and under the influence of climatic conditions. The total content of “core” components in the EOs of plants from “typical” habitats ranged from 48.31% to 92.60%, with an average value of 75.18%, while in the EOs of plants from “atypical” habitats it ranged from 4.82% to 77.17%, with an average value of 48.43%. The fluctuations in the content of camphor, 1,8-cineol, borneol and germacrene D were particularly noticeable.

**Figure 5:**
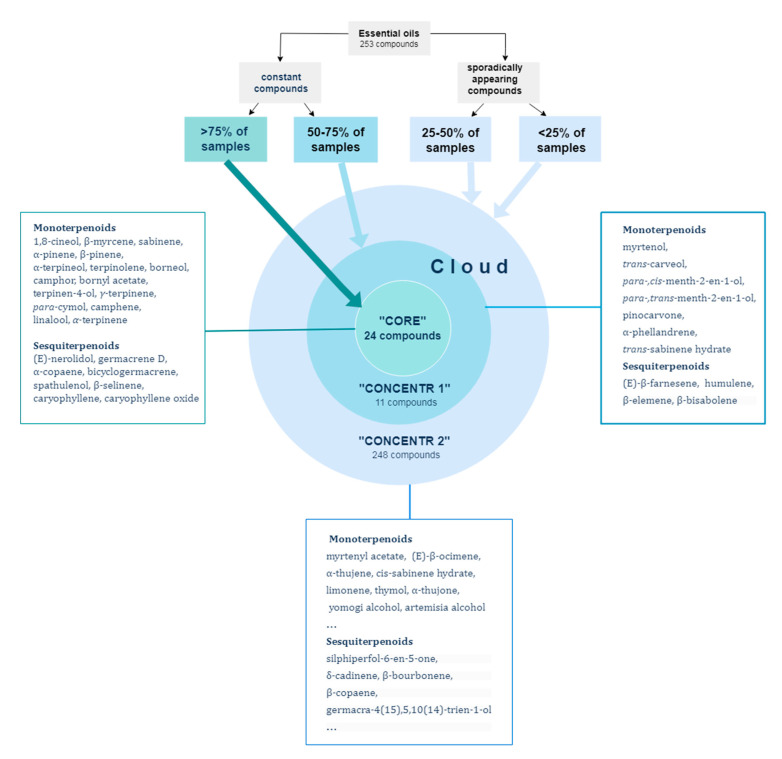
Conservative and labile components of essential oils of *A. frigida*.

Surrounding the “core” was a “cloud” of components whose formation was influenced by external factors. This “cloud” consisted of two concenters. The first concentric circle (11 compounds) included compounds that have been identified in samples from both groups but were found in the majority of essential oil samples from either “typical” or “atypical” habitats. The second concentric circle (248 compounds) comprised compounds that were found in a smaller number of samples from both groups (Figure 5). In addition to terpenoid compounds, the second concentric circle included phenylpropenes (eugenol, etc.) and acyclic compounds (capillene, etc.).

The conservatism of the qualitative composition of the “core” was largely related to the characteristics of the compounds’ biosynthesis. For example, one of the dominant components of *A. frigida* EOs, 1,8-cineol, was always accompanied by myrcene, limonene, sabinene, α-, β-pinene, α-terpineol [49], terpinolene, (E)-β-ocimene, and a-thujene [50,51] in small quantities, because they are formed under the action of the multiproduct enzyme cineole synthase. These components are known as “cineole cassette”.

Borneol and camphene are formed from a common precursor, bornyl diphosphate, and are catalyzed by the multiproduct enzyme BOR-synthases [52]. Other compounds, such as borneol and camphor, are interconnected. The intermediate bornyl diphosphate is hydrolyzed to borneol through the catalytic activity of bornyl diphosphate synthase and borneol synthase, and then borneol is oxidized to form camphor through the catalytic activity of bornyl dehydrogenase. Previously it was considered that the enzymatic reaction catalyzed by γ-terpinene synthase lead to the formation of γ-terpinene, which further transformed into p-cymene and the phenolic terpene isomers thymol and carvacrol [53]. Now it is established that γ-terpinene is oxidized by monooxygenases of the cytochrome P450 subfamily CYP71D to form unstable intermediate cyclohexadienols, which are then dehydrogenated by short-chain dehydrogenase/reductase (SDR) to the corresponding thymol and carvacrol. The formation of p-cymene occurs due to spontaneous rearrangement of the dienolic intermediate products because of their instability under aqueous conditions [54].

According to recent studies, both *2E,6E*-farnesyl pyrophosphate (*E,E*-FPP) and its isomer *Z,Z*-FPP are substrates for sesquiterpene synthases [55]. Acyclic sesquiterpenoids, such as (E)-β-farnesene and (E)-nerolidol, are formed through farnesyl and nerolidol cation, respectively. The biosynthesis of cyclic sesquiterpenoids is considered in terms of biogenetic pathways. In the essential oil of *A. frigida*, three groups (chemotypes) of sesquiterpenoids can be distinguished: germacrane, bisabolane and humulane sesquiterpenoids [56], with a predominance of germacrane compounds (Supplementary Materials, Table S7). The main compound of the germacrane chemotype in the EOs of *A. frigida* is germacrene D, which serves as a biogenetic precursor for many other sesquiterpene structures [57], including α-copaene, bicyclogermacrene, β-selinene and β-elemene. Spathulenol is formed from bicyclogermacrene during the extraction, storage and analysis of the essential oil [58]. Humulene, caryophyllene, caryophyllene oxide and triquinanes are representatives of the humulane series. All of these compounds were found in most samples and were part of the “core” and “concentric circle 1”. The exception was the group of triquinanes, which were found in specific samples. Among them, silphiperfol-6-en-5-one was the most frequently identified compound (48% of “typical” samples, and 3 out of 10 “atypical” samples). Bisabolane sesquiterpenoids were not represented in the “core”, and β-bisabolene was a part of “concentric circle 1”. The rest were included in “concentric circle 2”, but their content in the EOs of high-altitude plants was quite high. The total content of bisabolane sesquiterpenoids in samples from Qinghai province (China) ranged from 8.70% to 45.91%. “Concentric circle 2” included compounds formed as a result of further modification of terpenoids from the “core” and “concentric circle 1”. The presence of irregular monoterpenoids was observed in “concentric circle 2”, and they were formed through condensation of C5 units in a “non-head-to-tail orientation”, such as yomogi alcohol, lavandulol, artemisia alcohol and artemisia ketone.

Mono- and sesquiterpenoid compounds are multifunctional compounds. Their role in biotic interactions, such as allelochemicals, allelopathic compounds, antifeedants, attractants, and repellents, and their importance in plant defense against bacterial pathogens and fungi, has been well studied. At the same time, it is known that their emission is closely related to changes in abiotic factors in their environment. The existence of special biosynthetic pathways and the preservation of their emission, despite their “expensive” cost for plants (1–2% of photosynthetic carbon fixation) has led to the proposal of a model of a “unified biochemical mechanism for multiple physiological stressors”. It has been shown that volatile isoprenoids participate in direct reactions with oxidants and can also modify the transfer of ROS signals and/or contribute to membrane stabilization [4]. For example, α-bisabolol demonstrates high antioxidant activity in vitro [59]. Overall, the role of isoprene in plant thermotolerance has been extensively studied, while the role of volatile terpenoids has been less explored [60]. It has been shown that terpenes can protect plants from heat damage, and there is a correlation between monoterpene emission and heat stress resistance [61]. In our opinion, the emission of terpenoids by *A. frigida* explains its thermotolerance. The remarkable fact is that the “core” predominantly consists of compounds (pinene, 1,8-cineol, camphor, spatulenol, etc.) with pronounced allelopathic activity [20]. At the same time, β-caryophyllene is involved in attracting natural enemies of herbivorous insects [62] and in the defense against microbial pathogens [63].

The components of “concentric circle 1” can act as allelochemicals (para-menth-2-en-1-ol) or can have other activities. For example, (E)-β-farnesene on one hand is secreted by aphids as a signaling pheromone, and on the other hand it is produced by infected plants to attract natural enemies of aphids [64].

On the other hand, the composition of compounds in “concentric circle 2” is determined by the conditions of specific plant habitats, including abiotic factors and interactions with other plants, microorganisms, insects and herbivores, and even humans. Pathogen attack can shift biosynthesis towards the production of sesquiterpenoid phytoalexins [65]. Triquinanes have antibiotic properties [66]. The increase in the content of copaene in EOs, an intermediate compound in the pathway of terpenoid alkaloids’ biosynthesis, may also be associated with protection against mammals [67].

Changes in the content of compounds in EOs are associated with their emission. In high-altitude conditions, for example, there seems to be a faster conversion of borneol into camphor, resulting in a tendency for a decrease in borneol content and an increase in camphor content in the essential oil. Since we extract a pool of accumulated terpenoids from the tissues in the form of essential oil, we cannot definitively say whether there is an enhancement of camphor and borneol emission or if their biosynthesis is blocked at very high altitudes (4333 m above sea level). It seems that the biosynthetic pathways of the “core” components are maintained regardless of plant habitat conditions, while the rate of their emission changes. Specific factors, often biotic, influencing plants, affect the components of “concentric circle 1 and 2”.

Thus, mono- and sesquiterpenoids serve as the first line of defense for plants against biotic and abiotic stresses. They are essential for plant survival, and their immense diversity is shaped by factors that have both a genetic and epigenetic nature. Overall, we can observe a notable change in the compositional profile of *A. frigida* EOs under the influence of climatic parameters.

## 3. Materials and Methods

### 3.1. Plant Materials

The plant material was collected in the natural steppe habitats with chestnut soils within the territory of Russia (Republic of Buryatia) from 2016 to 2021 (Table 4). The collected raw material was dried to an air-dry state (2 weeks). Herbarium specimens are stored in the Laboratory of the Chemistry of Natural Systems at Baikal Institute of Nature Management, Siberian Branch, Russian Academy of Sciences.

### 3.2. Essential Oil Analyses

#### 3.2.1. Isolation of Essential Oils

A total of 30~40 g of the powdered plant material was subjected to hydrodistillation for 3 h using a Clevenger-type collector apparatus. The essential oils extracted in this research were obtained from the aerial parts of the plant. All experiments were conducted in triplicate, and the results were reported based on the air-dry weight.

#### 3.2.2. GC-MS Analysis of Essential Oils

The composition of the EOs was determined using gas chromatography–mass spectrometry (GC-MS) analysis after obtaining EOs. An Agilent 6890 gas chromatograph (Agilent Technologies, Santa Clara, CA, USA) equipped with an HP 5973 quadrupole mass selective detector (Hewlett-Packard, Palo Alto, CA, USA) and an HP-5MS capillary column (30 m *×* 0.25 mm *×* 0.2 µm; Hewlett-Packard) was used. The analysis was conducted with an electron impact at 70 eV and with the electron multiplier set at 2200 V. Helium was used as the carrier gas at a flow rate of 1 mL/min. The temperature of the column was programmed to increase from 50 to 240 °C at a rate of 4 °C/min, with an initial hold of 5 min, and then from 240 to 280 °C at a rate of 20 °C/min, with a final hold of 5 min. The injector and detector temperatures were set at 280 and 250 °C, respectively. The purity of the helium gas used was 99.999%. The column pressure was set to 52.8 × 10^3^ Pa, and the split ratio was 60:1.

#### 3.2.3. Essential Oil Compound Identification

The identification of essential oil constituents was conducted by comparing their mass spectra with standards from the NIST and with calculated linear retention indices (RIs) found in the literature [58]. The RIs were obtained by co-injecting a mixture of linear C8-C20 hydrocarbons. Identification was considered valid when the computer matching with the mass spectral libraries had a probability of over 90%. The contents of the oil components were determined by their relative amounts (%) and expressed as a percentage peak area relative to the total peak area, based on the GC-MS analyses of the oils.

### 3.3. Climatic Conditions

To characterize the main climatic parameters, data provided by the All-Russia Research Institute of Hydrometeorological Information (World Data Center) were used. The downloaded data included air temperatures, daily precipitation amounts, monthly precipitation sums, monthly air temperatures, number of days with precipitation equal to or greater than 1 mm, average monthly relative air humidity, monthly sunlight hours and more. Additionally, integrated indicators such as Selyaninov’s hydrothermal coefficient (HTC), temperature–humidity extremeness coefficient (Cextr) and the Standardized Precipitation Evapotranspiration Index (SPEI) were calculated.

The HTC was estimated to assess the levels of heat and humidity in specific regions using the formula HTC = R × 10/Σt. In this equation, R represents the precipitation sum in millimeters for June–July at mean daily temperatures above +10 °C, and Σt denotes the sum of mean daily temperatures in degrees Celsius (°C) for the same period.

The C_extr_ coefficient was calculated to compare the plant habitat conditions across different years. This coefficient represents the ratio of the mean temperature value for a specific period to the total precipitation for the same period, using the formula C_extr_ = t °C/mm.

Several parameters were estimated, including the sum of daily temperatures (Σt_veg_) and the sum of precipitation (ΣR_veg_) from the start of the vegetative phase (May) until the plant harvesting. Additionally, the sum of daily temperatures (Σt_year_) and the sum of precipitation (ΣR_year_) from August of the previous year to August of the year of plant harvesting were calculated. The temperature–humidity extremeness coefficients were estimated for both the period from the start of the vegetative phase until plant harvesting (C_extr.veg_), and for the entire year (C_extr.year_)_._ To simplify the calculations, the resulting values were multiplied by 100 and 1000, respectively, and rounded to whole numbers.

The Standardized Precipitation Evapotranspiration Index (SPEI) was calculated using the SPEI package. This index was estimated for both the calendar year (January–December) (SPEI_cal_) and the period from August of the previous year until August of the year of plant harvesting (SPEI_year_).

### 3.4. Statistical Analysis

To determine the influence of the main climatic parameters (air temperature, atmospheric precipitation, etc.) and their integral characteristics on the composition of plant essential oil, two types of linear regression models were constructed [68]. The first type of model was represented by the equation y = a*x + b. This type of model assumed that the content of a certain component of the essential oil, *y*, was linearly dependent on a certain parameter, *x*. The equation for the second type of model was z = a*x + b*y + c. Similar to the first case, a linear dependence was assumed between the content of a certain component of the essential oil, *y*, but this time on two parameters. The modeling was performed using the R programming language interpreter version 4.2.1 for the x86_64-pc-linux-gnu platform. The linear regression models were built using built-in R functions such as lm function and operations on data structures of the data.frame type. The Gauss–Markov hypothesis testing was conducted using the gvlma function from the “gvlma” package [69]. Correlation analysis was performed using the Excel2016 software package. For visualization purposes the data of the component composition of the essential oil were processed using principal component analysis (PCA) (Sirius version 6.0 software package, Pattern Recognition Systems, a/s, Bergen, Norway).

## 4. Conclusions

Essential oils are an important part of a plant’s protective system. The components of EOs serve as the first line of defense, primarily protecting plants against high temperatures. The component composition of EOs is most responsive to biotic interactions. Any stress triggers the activation of an antioxidant defense system in plants. The antioxidant properties of EOs are well known and undisputed [70].

The fluctuations in climatic parameters and their integral characteristics have no substantial influence on the ratio of components in the essential oil (chromatographic profile). Throughout its range, the composition of the essential oil remains similar, ensuring the best adaptation of plants to their growing conditions, and allowing *A. frigida* to exist as a dominant and co-dominant species in steppe vegetation. Noticeable changes in the component composition of *A. frigida’s* essential oil are associated with significant variations in the surrounding environment’s climatic parameters due to the confinement of *A. frigida* habitats to high mountains (over 1300 m a.s.l.) and/or extrazonal habitats (insolated slopes), as well as anthropogenic impacts (nearby roads).

The EOs of *A. frigida* consist of a “core” of constant components, totaling 24 compounds, as well as first and second “concentric circles” (11 and 248 compounds, respectively), which include variable components. Both the components of the “core” and the “concentric circles” exhibit variability in their mass fraction in the essential oil composition, due to the aforementioned external factors. It can be assumed that the biosynthesis of the components in the “core” is genetically controlled, while the biosynthesis of the compounds in the “concentric circles” is determined by epigenetic mechanisms. Genetic factors obviously play a significant role in the formation of the essential oil composition. Within this framework, changes in its composition are observed during ontogenetic development (at least in the latent and active periods). Variations in the composition also occur during seasonal development, with four patterns of composition changes observed in different phenophases. Additionally, the meteorological conditions of a specific vegetative period can influence the EO composition.

## Figures and Tables

**Table 1:** Chemical composition of EOs obtained from the seeds of *A. frigida* (Russia, Buryatia, Ivolginsky district, steppe. 15 September 1999).

RI *	Component	Peak Area (%)	RI *	Component	Peak Area (%)
932	pinene, *α*-	0.77	1162	pinocarvone	0.24
947	camphene	1.44	1166	borneol	22.46
975	pinene, *β*-	0.38	1177	terpinen-4-ol	4.79
991	myrcene, *β*-	1.13	1191	terpineol, *α*	1.81
1017	terpinene, *α*-	0.73	1287	bornyl acetate	3.57
1024	cymol, *para*-	0.63	1378	copaene, *α*-	0.22
1028	limonene	0.05	1387	bourbonene, *β*-	0.13
1031	cineol, 1,8-	25.11	1392	elemene, *β*-	0.10
1058	terpinene, *γ*-	1.33	1422	caryophyllene	0.36
1066	sabinene hydrate, *trans*-	1.44	1484	germacrene D	0.48
1088	terpinolene	0.28	1488	selinene, *β*-	1.18
1098	sabinene hydrate, *cis*-	1.08	1527	cadinene, *δ*-	0.23
1100	libalool	0.88	1580	spathulenol	0.76
1121	menth-2-en-1-ol., *para*-, *cis*-	0.43	1586	caryophyllene oxide	0.56
1144	camphor	22.58		*∑monoterpenes*	91.33
*∑sesquiterpenes*					4.02

* RI, retention indices: experimental, retention index as determined on HP-5MS column using the homologous series of n-hydrocarbons.

**Table 2:** Content of components in the essential oil of *Artemisia frigida* Willd. plants growing in the Ivolginsky, Selenginsky and Eravninsky districts of the Republic of Buryatia, according to linear regression models.

x	a	Y	b	c	z	*p*-Value	R2 (adjR2)
model z = a*x + b							
K_extr.year_	30.13		0.10		*trans-*carveol	0.001	0.62
model z = a*x + b*y + c							
∑R_June–August_	−0.002	∑t_veg._	−3.89 × 10^−4^	1.17	menth-2-en-1-ol, para-, trans-	6.70 × 10^−3^	0.52
Σt_June–August_	0.08	∑R_May–June_	0.77	−155.63	camphor	7.30 × 10^−4^	0.68
∑R_June–August_	0.03	Σt_June–August_	1.14 × 10^−2^	−18.32	bornyl acetate	2.91 × 10^−3^	0.59

**Table 3:** Hydrothermal coefficient of Selyaninov of Western and Eastern Siberia, 1999–2021 (May–August).

Year of Collection	Meteorological Station	Σt	R	HTC
Western Siberia				
1999	36038 Zmeinogorsk	2208.6	157.1	0.7
	36229 Ust-Kosa	1818.8	279.5	1.5
2000	29915 Slavgorod	2178.9	210.1	1.0
2001	36259 Kosh-Agach	1532.1	68.2	0.4
2003	360555 Kyzyl-Ozyok	1951.1	268.0	1.4
2003	36045 Soloneshnoye	2116.7	258.7	1.2
2005	29915 Slavgorod	2297.1	123.8	0.5
2007	36177 Semey	2341.9	181.3	0.8
2006	36096 Kyzyl	1884.7	193.7	1.0
Eastern Siberia				
2006	36307 Erzin	1829.8	96.7	0.5
2006	29862 Abakan	1836.5	212.3	1.2
1999	30636 Barguzin	1890.2	238.5	1.3
2014	30859 Aginskoye	1954.6	96.6	0.5
2014	30825 Ivolginsk	1881.0	97.9	0.5
2015	30745 Sosnovo-Ozerskoye	1708.3	175.9	1.0
2017	30632 Bolshoy Ushkany Island	1325.1	87.2	0.7
2017	30925 Kyakhta	2150.6	232.5	1.0
2017	30823 Ulan-Ude	2116.2	62.8	0.3
2021	30823 Ulan-Ude	1874.5	258.8	1.4

**Table 4:** Collection information for *Artemisia frigida* used in this study for the years 2016–2021.

Sample Code	Collection Date	Location	Latitude Longitude	Altitude (m)	Yield V/M (%)
BURi 12.08.2016	12 August 2016	Russia, Buryatia, Ivolginsky district, steppe	N 51°42 E 107°12	645	0.7
BURi 01.07.2017	1 July 2017				0.6
BURi 30.08.2017	30 August 2017				0.7
BURi 22.08.2018	22 August 2018				1.0
BURi 08.08.2020	8 August 2020				1.0
BURi 26.08.2020	26 August 2020				0.7
BURi 27.07.2021	27 July 2021				0.3
BURi 20.08.2021	20 August 2021				0.3
BURi 17.09.2021	17 September 2021				0.3
BURs 24.08.2016	24 August 2016	Russia, Buryatia, Selenginsky district, steppe	N 51°22 E 106°32	618	0.3
BURs 30.08.2017	30 August 2017				0.9
BURe 24.08.2017	24 August 2017	Russia, Buryatia, Eravninsky district, steppe	N 47°47 E 107°13	926	0.3
BURe 25.08.2016	25 August 2016				0.3
BURroad 19.08.2021	19 August 2021	Russia, Buryatia, Ivolginsky district, road	N 51°51 E 105°30	506	0.3
BURroad 17.09.2021	17 September 2021				0.3
BURhill 27.07.2021	27 July 2021	Russia, Buryatia, Ivolginsky district, the hill top	N 51°51 E 105°30	549	0.3
BURhill 19.08.2021	19 August 2021				0.3
BUR 1343 m 17.08.2016	17 August 2016	Russia, Buryatia, Okinsky district, steppe	N 33°21 E 96°58	1343	0.3
BURss 24.08.2016	24 August 2016	Russia, Buryatia, Selenginsky district, southern slope	N 51°26 E 106°34	689	0.3
BUR 27.08.2020	27 August 2020	Russia, Buryatia, Khorinsky district, steppe	N 52°13 E 109°57	772	0.7
BUR 08.07.2017	8 July 2017	Russia, Buryatia, Zaigraevsky district, steppe	N 52°27 E 108°71	575	0.3
BUR 31.08.2017	31 August 2017	Russia, Buryatia, Selenginsky district, steppe	N 51°26 E 106°34		0.3
BUR 12.08.2017	2 August 2017	Russia, Buryatia, Zaigraevsky district, steppe	N 51°42 E 107°12	572	0.7
OLNp 05.08.2017	5 August 2017	Russia, Irkytskaya oblast, Olkhon district, steppe	N 53°02 E 106°93	469	0.9
OLNs 05.08.2017	5 August 2017	Russia, Irkutskaya oblast, Olkhon district, vicinity of the Sokhor cove, steppe	N 53°02 E 106°76	491	1.0
OLNsh 05.08.2021	5 August 2021	Russia, Irkutskaya oblast, Olkhon district, vicinity of the Aya cove, steppe	N 52°79 E 106°60	780	0.7
CHN, 2016 4333 m	15 July 2016	China, Qinghai Province	N 33°08 E 96°43	4333	1.0

## Data Availability

All data generated or analyzed during this study are included in this published article.

## Supplementary Materials

The following supporting information can be downloaded at: https://www.mdpi.com/article/10.3390/plants12193422/s1, Table S1: Chemical composition of EOs obtained from the aerial parts of *A. frigida* from Russia and China (2016–2021). Table S2: Chemical composition of EOs obtained from the aerial parts of *A. frigida* from Russia and China (2016–2021) sorted by groups. Table S3: Chemical composition of EOs obtained from the aerial parts of *A. frigida* from Russia and China (2016–2021) sorted by groups in different phenological phases. Table S4: Values of climatic parameters in plant growing locations. Table S5: Results of Pearson’s linear correlation coefficient calculations between values of climatic parameters and components of essential oils (Ivolginsky district, July–September, 2021). Table S6: Constant compounds of *A. frigida* essential oils. Table S7: Content of acyclic and cyclic sesquiterpenoids, according to biogenetic trees.
